# Successful Treatment of* Clostridium difficile* Bacteremia with Aortic Mycotic Aneurysm in a Patient with Prior Endovascular Aortic Aneurysm Repair

**DOI:** 10.1155/2017/8472930

**Published:** 2017-02-27

**Authors:** Kairav Shah, Rebecca Brauch, Kartikeya Cherabuddi

**Affiliations:** Division of Infectious Diseases and Global Medicine, Department of Medicine, University of Florida, College of Medicine, 1600 SW Archer Rd., Gainesville, FL 32610, USA

## Abstract

The clinical spectrum of* Clostridium difficile *infection can range from benign gastrointestinal colonization to mild diarrhea and life threatening conditions such as pseudomembranous colitis and toxic megacolon. Extraintestinal manifestations of* C. difficile* are rare. Here, we report a patient with a history of an endovascular aortic aneurysm repair (EVAR) presenting with an endovascular leak complicated by* C. difficile* bacteremia and a mycotic aneurysm. He was successfully treated with an explant of the EVAR, an aorto-left renal bypass, and aorto-bi-iliac bypass graft placement along with a six-week duration of intravenous vancomycin and oral metronidazole.

## 1. Background

The clinical spectrum of* Clostridium difficile* infection (CDI) can range from benign gastrointestinal colonization to mild diarrhea and life threatening conditions such as pseudomembranous colitis and toxic megacolon. Extraintestinal manifestations of* C. difficile* are rare, 0.17% of all* C. difficile* infections [1]. These include bacteremia, perianal abscess, wound infection, and urinary catheter colonization. When associated with bacteremia, the infection is commonly polymicrobial and therapy has been directed at all organisms [2]. The case presented here is, to the best of our knowledge, the third reported case of* C. difficile* associated mycotic aneurysm.

## 2. Initial Presentation and Hospital Course

The patient is a 52-year-old man with past medical history of congestive heart failure, hypertension, alcohol abuse, and endovascular aneurysm repair (EVAR) 4 years previously who was admitted to an outside hospital with complaints of abdominal pain and diarrhea. A computed tomography (CT) of the abdomen revealed an expanding abdominal aortic aneurysm (AAA) with a type II endovascular leak (a type II leak refers to the presence of blood flow in the cavity of the aneurysm. These are associated with increased pressure within the side branches of the aorta leading blood to leak into the lower pressure aneurysm sac). This was treated endovascularly with embolization and thrombosis of the inferior mesenteric artery obliterating the type II leak. Patient was subsequently discharged home.

**Figure 1:**
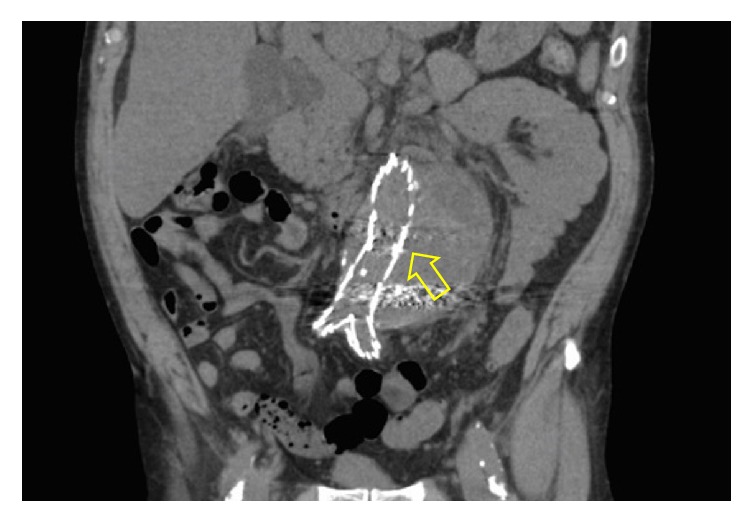
CT abdomen showing an expanding abdominal aortic aneurysm with few gas bubbles around prior endovascular graft (arrow).

One month later, he was readmitted due to continued abdominal pain. A follow-up CT was obtained and showed an expanding abdominal aortic aneurysm with few gas bubbles around prior endovascular graft concerning for infection (Figure 1), along with a rise in his white blood cell count to 14,000/mm^3^ (normal range: 4,000–10,000/mm^3^). This finding prompted transfer to our institute, a tertiary care referral center. On day 1 of admission, two sets of blood cultures were drawn. Patient was empirically started on intravenous (IV) piperacillin-tazobactam and vancomycin. Vascular surgery recommended that he undergo an EVAR conversion given the ongoing abdominal pain, enlarging aneurysm, and perianeurysmal air bubbles. On day 2, the patient underwent debridement, explant of the EVAR, aorto-left renal bypass with a rifampin soaked Dacron graft, and aorto-bi-iliac bypass with a rifampin soaked Dacron graft. Intraoperatively, it was noted that there was “a very inflamed aorta and purulent material was found.”

On day 3, blood cultures in two sets of anaerobic culture bottles grew Gram-positive rods. On day 5, the organism growing was identified as* C. difficile*. Aerobic blood culture bottles had no growth at 5 days. Two intraoperative aortic tissue cultures also grew* C. difficile*.

### 2.1. Microbiologic Methods

The Bactec 9240 automated blood culture system (Becton Dickinson Diagnostic Systems, Sparks, MD) was used to process aerobic and anaerobic blood culture bottles. Positive blood culture bottle specimens and intraoperative tissue samples from the abdomen and the aorta were plated in the anaerobic chamber on Brucella Blood Agar, Anaerobe Systems. Once growth occurred, the inoculum along with RapID™ Inoculation Fluid was poured into wells in the Thermo Scientific™ RapID ANA II System, an identification system used for anaerobes that utilizes a qualitative micromethod employing conventional and chromogenic substrates. Identification of* C. difficile* was made based on comparison of reactivity score to a RapID ANA II differential chart.

The method currently recommended by the Clinical and Laboratory Standards Institute (CLSI) for* C. difficile* antimicrobial susceptibility testing is the agar dilution method using supplemented Brucella agar [3]. The agar dilution method is very technically demanding and labor-intensive and is difficult to implement for routine use in the clinical laboratory and therefore was not performed. Disk diffusion and/or E-test (Epsilometer test) are easier to perform but no CLSI standardization or guidelines have been established for* C. difficile *[3]. Genotyping of the isolate was not performed.

### 2.2. Outcome and Follow-Up

Since there were Dacron grafts in critical vascular areas and paucity of literature on treating intravascular infections with* C. difficile*, his antibiotic regimen was changed to IV vancomycin and IV metronidazole on day 5 of his hospital stay. Piperacillin-tazobactam was discontinued. A result of stool* C. difficile* PCR test done on day 6 was negative. He was finally discharged from the hospital on postoperative day 10 with a six-week treatment duration of IV vancomycin and oral metronidazole.

One month after discharge, a screening colonoscopy did not reveal any aortoenteric fistula or colorectal malignancy. Follow-up CT imaging at two months (at the end of antibiotic therapy) and at seven months after surgery showed stability of the aorta and grafts. He recovered well with no other complications or relapse of the bacteremia. Suppressive antimicrobial therapy after the initial course was not done due to concerns of long-term neurotoxicity from metronidazole use and lack of evidence to support it in this rare case.

## 3. Discussion

Upon review of the literature, the consensus is that* C. difficile* bacteremia and extraintestinal manifestations are rare but when found they are associated with a rather high mortality rate, up to 36% in one review [1]. Additionally, they are associated with hospitalized patients with comorbidities and recent antibiotic exposure, and frequently the bacteremia is polymicrobial [1, 2].

The differential diagnosis for periaortic emphysema is either an aortoenteric fistula or mycotic aneurysm from a gas producing organism. Intravascular infections and mycotic aortic aneurysms have been well described in* Clostridium* spp., especially with* Clostridium septicum* and the strong association with occult colonic adenocarcinoma [4]. The aorta is thought to be seeded from bacteremia resulting from disruption of the colonic wall by the adenocarcinoma. Interestingly,* C. septicum *disseminates uniquely and very well to the site of the soft tissue infection in nontraumatic myonecrosis or to the aorta causing aortic aneurysm [5]. This may be secondary to the presence of flagellar motility and endotoxins. The alpha toxin has been described as essential to its virulence [6].* C. septicum* is invasive and also has been shown to impair neutrophil function.

In contrast,* C. difficile* bacteremia (CDB) is a rare event even in the presence of epidemic levels of colitis with CDI. The CDB cases described are anecdotal and sporadic. In the largest such series of 12 cases over a 10-year-period and 20 other reported cases [7], most patients had gastrointestinal complaints or conditions but less than half had diarrhea. In contrast to* C. septicum* bacteremia, most cases with* C. difficile* were polymicrobial bacteremia (so cannot be attributed just to* C. difficile* virulence alone). Only 64% of 22 cases had either toxin A or toxin B.* C. difficile* is not considered invasive even though the toxin is potent at causing local edema and eliciting a robust inflammatory response. These factors likely explain the paucity of bacteremia with* C. difficile*.

The mechanism of bacteremia with* C. difficile* is presumed to be due to colonic wall inflammation associated with pseudomembranous colitis, perforation, or surgery that leads to a transient bacteremia [1, 8]. It is possible that an anaerobic thrombus promotes the survival of* C. difficile* in the aneurysm [1]. A case report of* C. difficile* mycotic aneurysm involving the iliac artery by Tsukioka et al. noted that a colonoscopy performed after resolution of initial septic shock revealed pseudomembranous colitis, but stool toxin was negative. They suspected that transient bacteremia (blood cultures were negative for* C. difficile*) due to pseudomembranous colitis led to infection of the iliac artery [9]. Additionally, aortitis and endovascular infections have been reported as complications after coil embolization of aortic side branches [10].

Our case is the third reported case of* C. difficile *causing a mycotic aneurysm to the best of our knowledge; the other two cases involved the iliac artery [9] and the aorta [11]. We hypothesize that embolization of the inferior mesenteric artery in the setting of diarrhea during the patient's first hospitalization could have led to development of a transient bacteremia which then led to aortitis and a mycotic aneurysm. Upon review of the outside hospital records, it did not appear that a stool test for* C. difficile* had been performed during his previous hospitalization. A stool for* C. difficile *PCR was negative at our facility; however, the patient did not have active diarrhea at that time and he had already received IV piperacillin-tazobactam and IV vancomycin as well as IV metronidazole for a few days prior to testing, drugs with activity against* C. difficile*.

Treatment of* C. difficile* endovascular infections is not well defined. Resistance to vancomycin has only been sporadically reported and 97.8% of* C. difficile* isolates were shown to be susceptible with the majority of the nonsusceptible ones having intermediate resistance and only a few resistant [12, 13]. These have been described more so from Europe. Resistance to metronidazole was reported to be only 0.11% in the same study [12]. Even with reports of decreased susceptibility and only moderate fecal concentration there is no link between metronidazole resistance in* C. difficile* and treatment failure [13, 14]. Rifamycins have activity on* C. difficile* and rifaximin is used as an adjunct in treatment of CDI. Resistance to rifampin has been reported to be from 0 to 17.5% depending on the study [13]. While the Dacron grafts placed were rifampin soaked, parenteral rifampin was not used due to concern for tolerability and the grafts being placed after resection of infected tissue.

In conclusion,* C. difficile* bacteremia and aortic aneurysms are rare events and not known to be associated with malignancy in contrast to* C. septicum*. The correlation with* C. difficile* associated diarrhea and bacteremia is weak and is probably secondary to the noninvasive nature of* C. difficile*. Early diagnosis, surgical management, and long courses of appropriate antibiotics are indicated in vascular infections with* C. difficile*. Suppressive antibiotic therapy and use of rifampin need to be tailored to individual patients. The literature supports aggressive treatment of* C. difficile* associated mycotic aneurysms due to the high mortality rate [15].
